# The ALK inhibitor ASP3026 eradicates NPM-ALK^+^ T-cell anaplastic large-cell lymphoma *in vitro* and in a systemic xenograft lymphoma model

**DOI:** 10.18632/oncotarget.2170

**Published:** 2014-07-05

**Authors:** Suraj Konnath George, Deeksha Vishwamitra, Roxsan Manshouri, Ping Shi, Hesham M. Amin

**Affiliations:** ^1^ Department of Hematopathology, The University of Texas M. D. Anderson Cancer Center, Houston, TX; ^2^ The University of Texas Graduate School of Biomedical Sciences, Houston, TX; ^3^ State Key Laboratory of Bioreactor Engineering, East China University of Science and Technology, Shanghai, China

**Keywords:** NPM-ALK, ASP3026, T-cell lymphoma, crizotinib, CHOP

## Abstract

NPM-ALK^+^ T-cell anaplastic large-cell lymphoma (ALCL) is an aggressive type of cancer. Standard treatment of NPM-ALK^+^ ALCL is CHOP polychemotherapy. Although patients initially respond favorably to CHOP, resistance, relapse, and death frequently occur. Recently, selective targeting of ALK has emerged as an alternative therapeutic strategy. ASP3026 is a second-generation ALK inhibitor that can overcome crizotinib resistance in non-small cell lung cancer, and is currently being evaluated in clinical trials of patients with ALK^+^ solid tumors. However, NPM-ALK^+^ ALCL patients are not included in these trials. We studied the effects of ASP3026 on NPM-ALK^+^ ALCL cell lines *in vitro* and on systemic lymphoma growth *in vivo*. ASP3026 decreased the viability, proliferation, and colony formation, as well as induced apoptotic cell death of NPM-ALK^+^ ALCL cells. In addition, ASP3026 significantly reduced the proliferation of 293T cells transfected with NPM-ALK mutants that are resistant to crizotinib and downregulated tyrosine phosphorylation of these mutants. Moreover, ASP3026 abrogated systemic NPM-ALK^+^ ALCL growth in mice. Importantly, the survival of ASP3026-treated mice was superior to that of control and CHOP-treated mice. Our data suggest that ASP3026 is an effective treatment for NPM-ALK^+^ ALCL, and support the enrollment of patients with this lymphoma in the ongoing clinical trials.

## INTRODUCTION

Anaplastic lymphoma kinase (ALK) is a receptor tyrosine kinase that is expressed in neuronal cells at an early stage of human development; therefore, it is assumed that ALK contributes to neural tissue development [1, 2]. In addition to its physiologic expression, ALK is aberrantly expressed in several human cancers, including an aggressive subtype of T-cell lymphoma known as ALK-expressing anaplastic large-cell lymphoma (ALK^+^ ALCL) [3]. The expression of ALK in this lymphoma occurs secondary to one of several chromosomal aberrations that involve the *ALK* gene on chromosome 2p23. The most common of these aberrations is translocation t(2;5)(p23;q35) that occurs in 85% of ALK^+^ ALCL cases [3, 4]. This translocation also involves the nucleophosmin (*NPM*) gene and induces the generation of the oncogenic tyrosine kinase NPM-ALK.

NPM is a ubiquitously expressed protein that shuttles ribonucleoproteins between the nucleolus and cytoplasm. Unlike ALK, NPM-ALK lacks an extracellular domain. Nonetheless, NPM-ALK appears to be constitutively activated by the formation of the NPM-ALK/NPM-ALK homodimers through the NPM domain. Wild-type NPM is also involved in the formation of NPM/NPM-ALK heterodimers that translocate to the nucleus [5]. Because NPM-ALK is the most widely expressed ALK chimeric protein and because all established ALK^+^ T-cell ALCL cell lines harbor NPM-ALK, in this paper we will refer to this lymphoma as NPM-ALK^+^ ALCL.

Although NPM-ALK^+^ ALCL represents only 3-5% of non-Hodgkin lymphomas in adults, it constitutes up to 40% of these lymphomas in children and adolescents. NPM-ALK^+^ ALCL is an aggressive cancer; most patients present with advanced stage (III/IV) disease, widespread dissemination, and B symptoms including high fever, night sweats, and weight loss [3]. There is no specific therapeutic approach for NPM-ALK^+^ ALCL, and it is typically treated with doxorubicin-containing polychemotherapy such as CHOP (cyclophosphamide, doxorubicin, vincristine, and prednisone). A few studies have systematically analyzed the outcomes of therapeutic approaches for NPM-ALK^+^ ALCL, and, not unexpectedly, most of these studies focused on pediatric patients [6-13]. Patients usually show an initial favorable response to CHOP; however, 30-40% of the patients in these studies had multiple relapses and disease/therapy-associated death remains a common event [7, 12].

Because ALK plays crucial roles in the development and survival of NPM-ALK^+^ ALCL [14], targeting ALK is viewed as a promising strategy for treating NPM-ALK^+^ ALCL patients [15]. Although selective ALK inhibitors are being evaluated in clinical trials, most of these trials focus on ALK^+^ solid tumors such as non-small cell lung cancer (NSCLC), neuroblastoma, and inflammatory myofibroblastic tumor [16-18].

ASP3026 (2-N-[2-methoxy-4-[4-(4-methylpiperazin-1-yl)piperidin-1-yl]phenyl]-4-N-(2-propan-2-ylsulfonylphenyl)-1,3,5-triazine-2,4-diamine) is a recently developed, orally available, second-generation small molecule ALK inhibitor. ASP3026 was identified in an ALK inhibition assay aimed at the oncogenic fusion kinase echinoderm-microtubule-associated protein-like 4-ALK (EML4-ALK) that has been detected in a subgroup of NSCLC patients [19, 20]. Initial studies showed that ASP3026 possesses remarkable inhibitory effects on EML4-ALK activity. Preliminary studies in NSCLC showed that compared with crizotinib (PF-02341066, Xalkori), an ALK/c-MET/ROS1 inhibitor that has been approved to treat patients with relapsed ALK^+^ NSCLC, ASP3026 is more tolerable and more potent in inhibiting EML4-ALK [20]. Importantly, crizotinib-resistant EML4-ALK mutants were not resistant to the effects of ASP3026 [20-23].

Because of these encouraging results, ASP3026 is currently being evaluated in clinical trials (http://clinicaltrials.gov/show/NCT01401504; http://clinicaltrials.gov/ct2/show/NCT01284192) that enroll patients with aggressive ALK^+^ solid tumors and ALK^+^ large B-cell lymphoma, which, compared with NPM-ALK^+^ T-cell ALCL, is a very rare entity with less than 40 patients reported [24]. A preliminary analysis concluded that ASP3026 possesses promising safety and pharmacokinetic profiles [25]. Although NPM-ALK^+^ ALCL was the first type of cancer in which ALK was identified, the effects of ASP3026 have not been evaluated in this neoplasm. More importantly, NPM-ALK^+^ ALCL patients are not eligible for enrollment in the ongoing trials.

Herein, we sought to perform a systematic *in vitro* analysis of the effects of ASP3026 on NPM-ALK^+^ ALCL cells. In addition, we developed and utilized a systemic xenograft NPM-ALK^+^ ALCL model in SCID mice to examine the effects of this inhibitor *in vivo*. Our data provide strong evidence that ASP3026 is an efficacious treatment for the aggressive NPM-ALK^+^ ALCL, and support the inclusion of NPM-ALK^+^ ALCL patients in the ongoing clinical trials.

## RESULTS

### ASP3026 decreases NPM-ALK^+^ ALCL cell viability

**Figure 1:**
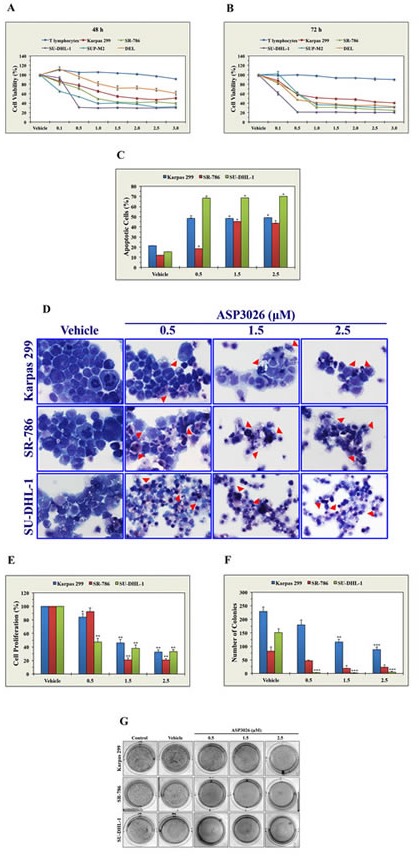
ASP3026 induces a pronounced negative biological impact in NPM-ALK^+^ ALCL cells (A) At 48 h, ASP3026 induced concentration-dependent decreases in the viability of NPM-ALK^+^ ALCL cells, as measured by MTS assay. These effects were not detected in T lymphocytes, and the decrease in the viability of NPM-ALK^+^ ALCL cells was statistically significant compared to T lymphocytes (at 0.1 μM concentration: *P* < 0.05 in SU-DHL-1, *P* < 0.01 in Karpas 299, *P* = 0.0001 in SR-786, *P* < 0.0001 in SUP-M2, *P* > 0.05 in DEL [not significant]; at 0.5 μM concentration: *P* < 0.0001 in all cell lines except DEL where P > 0.05 [not significant]; at ≥ 1.0 μM concentration: P < 0.0001 in all cell lines). (B) The effects of ASP3026 on cell viability were more pronounced at 72 h after treatment (at ≥ 0.5 μM: *P* < 0.0001 in all cell lines compared with T lymphocytes). (C) Treatment of the NPM-ALK^+^ ALCL cells Karpas 299, SR-786, and SU-DHL-1 with ASP3026 for 48 h was associated with significant concentration-dependent increases in apoptotic cell death, as measured by flow cytometry after cellular staining with annexin V/PI (*: *P* < 0.0001 compared with vehicle-treated control cells). (D) Morphologic features associated with apoptotic cell death, including cellular shrinkage and nuclear condensation and fragmentation, are shown in Karpas 299, SR-786 and SU-DHL-1 cells after treatment with ASP3026 for 48 h (red arrowheads). (E) ASP3026 also decreased the proliferation of NPM-ALK^+^ ALCL cells as measured by BrdU assay (*: *P* < 0.05, **: *P* < 0.0001 compared with control cells treated with vehicle). (F) Moreover, ASP3026 abrogates anchorage-independent colony formation of NPM-ALK^+^ ALCL cells in methylcellulose (*: *P* < 0.05, **: *P* < 0.01, ***: *P* < 0.001 compared with vehicle-treated control cells). (G) Representative cultures illustrating the marked decrease in the number of NPM-ALK^+^ ALCL cell colonies at 5 days after treatment with ASP3026 for 48 h. Results are shown as means ± SE of at least 3 consistent experiments.

At 48 h, ASP3026 induced concentration-dependent decreases in the viability of all NPM-ALK^+^ ALCL cell lines tested, as measured by MTS assay (Fig. 1A). DEL cells, particularly at lower concentrations of 0.1, 0.5, and 1.0 μM, demonstrated less sensitivity to ASP3026 than the other cell lines. The IC_50_ was 0.4 μM in SU-DHL-1, 0.75 in SUP-M2, 1.0 in SR-786, 2.5 in Karpas 299, and > 3.0 in DEL cell lines. Similar effects were not observed in the T lymphocytes that were used as a negative control and the decrease in the viability of the lymphoma cells was significantly greater than the decrease in the viability of the T lymphocytes. The decrease in cell viability became more pronounced at 72 h, and the IC_50_ was 0.3 μM in SU-DHL-1, 0.5 in DEL, 0.75 in SUP-M2 and SR-786, and 2.5 in Karpas 299 (Fig. 1B).

### ASP3026 induces apoptosis in NPM-ALK^+^ ALCL cells

Analysis of apoptosis by flow cytometry after dual staining with annexin V-FITC and PI showed that at 48 h ASP3026 (0.5, 1.5, and 2.5 μM) induced significant apoptosis in Karpas 299, SR-786, and SU-DHL-1 cells (Figure 1C). Apoptosis was also documented by Giemsa staining (Figure 1D).

### ASP3026 decreases the proliferation of NPM-ALK^+^ ALCL cells

Karpas 299, SR-786, and SU-DHL-1 cells were treated with ASP3026 and changes in cell proliferation were measured using a BrdU assay. At 48 h, concentration-dependent decreases in cell proliferation were noted in the 3 cell lines (Figure 1E).

### ASP3026 abrogates anchorage-independent colony formation of NPM-ALK^+^ ALCL cells

Figure 1F shows that treatment with ASP3026 for 48 h induced concentration-dependent decreases in colony numbers in Karpas 299, SR-786, and SU-DHL-1 cells grown for 5 days in methylcellulose. This experiment showed that SU-DHL-1 cells were most sensitive to the effects of ASP3026. Representative images of colonies from untreated cells, cells treated with vehicle, and cells treated with ASP3026 are shown in Figure 1G. Compared with cells treated with vehicle or untreated cells, NPM-ALK^+^ ALCL cells treated with ASP3026 developed remarkably fewer colonies.

### ASP3026 reduces the tyrosine kinase activity of NPM-ALK and its phosphorylation levels and induces significant alterations in downstream targets of NPM-ALK signaling

**Figure 2:**
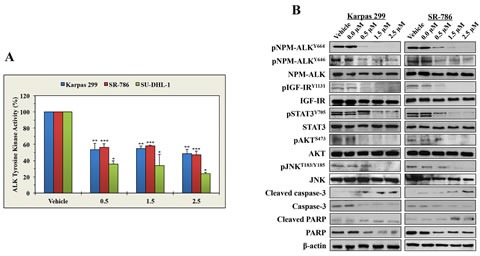
ASP3026 reduces the tyrosine kinase activity of NPM-ALK, downregulates the phosphorylation of NPM-ALK and target proteins, and induces biochemical effects consistent with apoptosis (A) ASP3026 induced a marked decrease in NPM-ALK tyrosine kinase activity in the lymphoma cells (*: *P* < 0.01, **: *P* < 0.001, ***: *P* < 0.0001 compared with control cells treated with vehicle). Results shown are means ± SE of 3 experiments. (B) ASP3026 decreased the phosphorylation of NPM-ALK at 2 different tyrosine residues: Y646 and Y664. Also, treatment with ASP3026 was associated with downregulation of the phosphorylation of the NPM-ALK target proteins IGF-IR, STAT3, AKT, and JNK. Changes in the basal levels of these proteins were not detected. The occurrence of apoptosis was biochemically supported by the increase in cleaved caspase-3 and cleaved PARP and the simultaneous decrease in non-cleaved caspase-3 and non-cleared PARP levels. β-Actin indicates equal loading of the proteins.

To seek an explanation for the negative biological impact of ASP3026 in NPM-ALK^+^ ALCL cells, we measured NPM-ALK tyrosine kinase activity and phosphorylation levels after treatment with ASP3026. ASP3026 induced marked decreases in the tyrosine kinase activity of NPM-ALK in Karpas 299, SR-786, and SU-DHL-1 cells (Figure 2A). Furthermore, Western blotting results showed that ASP3026 downregulated pNPM-ALK at Y646 and Y664 (Figure 2B). Moreover, ASP3026 induced remarkable downregulation of the phosphorylation of several survival-promoting proteins that are known to be activated by NPM-ALK including IGF-IR, STAT3, AKT, and JNK (Figure 2B). The occurrence of apoptosis after treatment with ASP3026 was biochemically supported by cleavage of caspase 3 and PARP (Figure 2B).

### ASP3026 decreases the proliferation of 293T cells transfected with crizotinib-resistant NPM-ALK mutants

**Figure 3:**
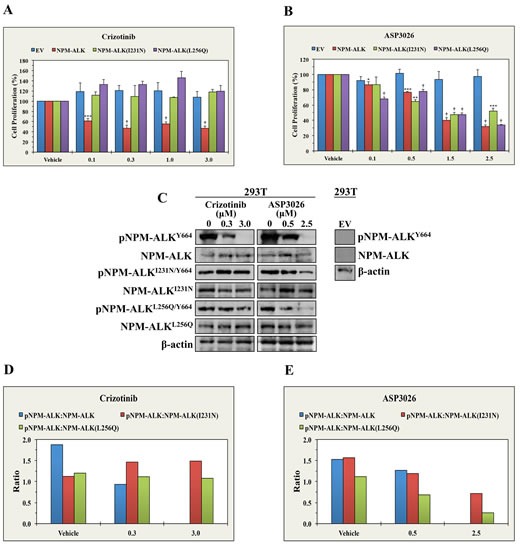
ASP3026 overcomes the resistance to crizotinib in NPM-ALK^+^ ALCL (A) Whereas crizotinib induced significant decrease in the proliferation of 293T cells transiently transfected with NPM-ALK, it failed to induce similar effects in the cells transfected with EV, NPM-ALK^I231N^ or NPM-ALK^L256Q^. (B) ASP3026 successfully decreased the proliferation of 293T cells transfected with NPM-ALK, NPM-ALK^I231N^ or NPM-ALK^L256Q^ (*: *P* < 0.05, **: *P* < 0.01, ***: *P* < 0.001, †: *P* < 0.0001). No effects were seen when control 293T cells transfected with EV were treated with ASP3026. Results represent the means ± SE of 3 experiments. (C) The left panel shows that crizotinib decreased the phosphorylation of NPM-ALK transfected in 293T cells, but not the phosphorylation of NPM-ALK^I231N^ or NPM-ALK^L256Q^. At the other hand, ASP3026 reduced the phosphorylation of the three constructs. The levels of non-phosphorylated NPM-ALK constructs and β-actin support equal protein loading. The right panel illustrates lack of NPM-ALK/pNPM-ALK protein expression in 293T cells transfected with EV. (D) Densitometric analysis depicting the ratio of pNPM-ALK Western blot bands to their corresponding NPM-ALK bands is shown. The results illustrate that crizotinib induced remarkable downregulation of pNPM-ALK in 293T cells transfected with NPM-ALK but not in 293T cells transfected with crizotinib-resistant NPM-ALK mutants. (E) Furthermore, the densitometric analysis also shows that treatment with ASP3026 caused concentration-dependent decrease in pNPM-ALK levels in cells transfected with NPM-ALK, NPM-ALK^I231N^ or NPM-ALK^L256Q^.

To test whether ASP3026 can overcome the resistance to crizotinib, we used crizotinib or ASP3026 to treat 293T cells transiently transfected with NPM-ALK, NPM-ALK^I231N^ or NPM-ALK^L256Q^. Not surprisingly, crizotinib (Figure 3A) and ASP3026 (Figure 3B) induced concentration-dependent decrease in the proliferation of cells transfected with NPM-ALK. Whereas ASP3026 efficiently decreased the proliferation of cells transfected with NPM-ALK^I231N^ or NPM-ALK^L256Q^, crizotinib failed to induce similar effects. To further analyze the differential effects of crizotinib and ASP3026, we studied the changes in pNPM-ALK levels. Indeed, crizotinib and ASP3026 caused marked downregulation of pNPM-ALK at Y664 in 293T cells transfected with NPM-ALK. Nonetheless, only ASP3026 was capable of reducing pNPM-ALK levels at Y664 in cells transfected with NPM-ALK^I231N^ or NPM-ALK^L256Q^ (Figure 3C). Densitometry of the Western blot bands is shown (Figures 3D and 3E).

### ASP3026 suppresses NPM-ALK^+^ ALCL tumor growth *in vivo*

**Figure 4:**
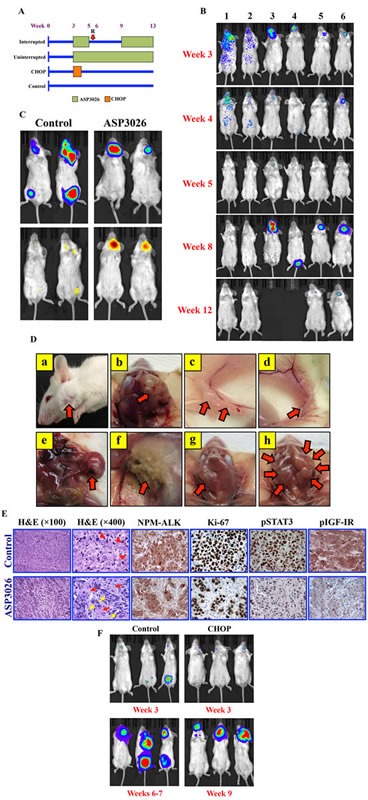
ASP3026 suppresses NPM-ALK^+^ ALCL tumor cell growth *in vivo* (A) C.B-17 SCID mice were randomized into the 4 illustrated treatment groups at week 3 after intravenous injection of Karpas 299 cells permanently expressing firefly luciferase. “R” denotes relapse after ASP3026 discontinuation. (B) Mice developed NPM-ALK^+^ ALCL tumors within approximately 3 weeks after injection, and were monitored weekly using bioluminescence imaging. The intensity of the signal is indicated by color (blue: low; green: intermediate; red: high tumor burden). Mice uninterruptedly treated with ASP3026 (examples shown in lanes 1 & 2) underwent complete remission with no relapse until study termination at week 13. Administration of ASP3026 was interrupted in 5 mice (examples in lanes 3-6) for 4 weeks. Notably, ALCL relapsed as early as 1 week after discontinuation of ASP3026. Two mice (lanes 3 & 4) had to be euthanized before the study period ended because of relapsed NPM-ALK^+^ ALCL with extensive tumor burden. At week 9, ASP3026 was re-administered in the other 3 mice (2 shown in lanes 5 & 6), and the NPM-ALK^+^ ALCL tumors substantially regressed, and the mice survived until termination of the study. (C) Bioluminescence imaging highlights the NPM-ALK^+^ ALCL tumors in control and ASP3026-treated mice (upper panels). Mice were injected intraperitoneally by the firefly luciferase prosubstrate VivoGlo Caspase-3/7 and bioluminescence imaging was performed. Tumors in control mice treated only with vehicle failed to demonstrate significant apoptosis signals (left lower panel). In contrast, intense signals consistent with marked *in vivo* lymphoma cell apoptosis were detected in mice treated with ASP3026 (right lower panel). (D) Examples of control mice showing lymphoma (red arrows) in cervical lymph nodes (a, exterior; b, dissected); inguinal lymph nodes (c, d); mesenteric lymph nodes (e); and axillary lymph nodes (f). Example of cervical lymph nodes is shown in a mouse treated interruptedly with ASP3026 (group I). After relapse, this mouse received ASP3026, survived, and was euthanized at the end of the study. A small cervical lymph node (red arrow) was detected at necropsy (g). This lymph node was processed and microscopically examined as shown in Figure 3E, lower panel. Example of cervical lymph nodes is shown after relapse in a mouse treated interruptedly with ASP3026. The lymph nodes comprised large, bulky tumor masses (red arrows) (h). (E) Histologic sections from cervical lymph node obtained from a control mouse treated with vehicle show effacement of the normal nodal architecture by solid sheets of lymphoma cells (H&E staining; original magnification: ×100). Higher magnification shows large cells with abundant cytoplasm, vesicular nuclei, and high mitotic rate with atypical mitotic figures [red arrowheads] (H&E; ×400). Immunohistochemical staining shows cytoplasmic and nuclear localization of NPM-ALK protein in the solid sheets of the lymphoma cells. Ki-67 is expressed in the vast majority of the lymphoma cells that diffusely infiltrate the lymph node. In addition, pSTAT3^Y705^ and pIGF-IR^Y1161^ are highly expressed in almost all of the lymphoma cells. Also shown are histologic sections from a small post-treatment cervical lymph node collected at necropsy from a mouse treated with ASP3026 after relapse (I group, illustrated in Figure 3D, panel g). H&E staining shows small nests of ALCL cells separated by thick stromal tissue strands (×100). Higher magnification of the same lymph node further illustrates the small clusters of lymphoma cells with rare mitotic figures [yellow arrows] separated by thick stromal strands that include fibroblasts, small blood vessels, and admixed inflammatory cells [red arrows] (×400). Although NPM-ALK and Ki-67 were identified in residual ALCL cells, the numbers of these cells were much less pronounced after treatment. The expressions of pSTAT3 and pIGF-IR are substantially reduced in the lymph node sections from the ASP3026-treated mouse. Immunohistochemical staining photomicrographs are shown at an original magnification of ×400. (F) Control mice at week 3 (top left) and weeks 6-7 (bottom left) show remarkable progression of systemic NPM-ALK^+^ ALCL. Examples of mice from the CHOP-treatment group with NPM-ALK^+^ ALCL established at week 3 (top right). Although CHOP initially suppressed NPM-ALK^+^ ALCL tumor growth, lymphoma relapsed as illustrated at week 9 and the mice had to be euthanized because of heavy tumor burden (bottom right).

Female C.B-17 SCID mice were randomized into 4 treatment groups (control [10 mice]; uninterrupted ASP3026 [U, 5]; interrupted ASP3026 [I, 5]; and CHOP [10]) at 3 weeks after intravenous injection of Karpas 299 cells permanently expressing firefly luciferase (Figure 4A).

Lymphoma tumor burden was monitored weekly using bioluminescence imaging and detection of signal intensity. ASP3026-treated mice demonstrated remarkable lymphoma regression at 2 weeks after treatment initiation. Mice treated uninterruptedly with ASP3026 demonstrated complete remission with no relapse or lymphoma-related death. Representative examples are shown in Figure 4B (lanes 1 & 2). Notably, ALCL relapsed as early as 1 week after interruption of ASP3026 (examples shown in Figure 4B; lanes 3-6). Two mice with relapse were euthanized at week 8 owing to heavy tumor burden (Figure 4B; lanes 3 & 4). At week 9, ASP3026 was re-administered to the other 3 mice; their tumors substantially regressed and they lived until study termination (examples shown in Figure 4B; lanes 5 & 6). The inhibitory effects of ASP3026 on NPM-ALK^+^ ALCL growth were attributable, at least in part, to *in vivo* apoptotic cell death (Figure 4C; right lane). Significant apoptosis was not observed in control mice treated with vehicle (Figure 4C; left lane). The occurrence of disseminated systemic lymphoma in cervical, inguinal, mesenteric, and axillary lymph nodes from control mice was confirmed at necropsy (Figure 4D, panels a-f). Figure 4D, panel g, shows a small cervical lymph node from a mouse from the interrupted (I) ASP3026 group that developed relapsed lymphoma and thereafter was treated with ASP3026 and survived until the termination of the study. Figure 4D, panel h, illustrates markedly enlarged cervical lymph nodes involved with lymphoma in a mouse from the interrupted ASP3026 group (I) after relapse. This mouse was euthanized before the end of the study because of heavy tumor burden.

Histologic examination of tissues obtained from control mice showed infiltration by solid sheets of malignant lymphoma cells (Figure 4E). Expression of ALK was confirmed by immunohistochemical analysis (Figure 4E). Ki-67 was expressed in the vast majority of the lymphoma cells that diffusely infiltrated the tissues. In addition, strong expressions of pSTAT3 and pIGF-IR were detected in most of the lymphoma cells (Figure 4E). Conversely, tissues from a mouse from the interrupted ASP3026 group (I) that was treated with ASP3026 after relapse (lymph node is shown in figure 4D, panel g) illustrated that treatment with ASP3026 was associated with marked decrease in the number of tumor cells. Instead of the solid ALCL sheets seen in control lymph nodes; after treatment with ASP3026, scattered nests of ALCL cells separated by thick stromal strands were detected (Figure 4E). Although ALK and Ki-67 were present in residual ALCL cells in the treated tumors, their numbers were much lower. Moreover, the expression of pSTAT3 and pIGF-IR were markedly decreased after treatment with ASP3026 (Figure 4E).

In another group, NPM-ALK^+^ ALCL tumor-bearing mice were treated with CHOP. Although CHOP initially suppressed NPM-ALK^+^ ALCL tumor growth, aggressive lymphoma later relapsed and the mice had to be euthanatized before completion of the study because of heavy tumor burden. Figure 4F shows examples of control mice and mice treated with CHOP.

### Treatment with ASP3026 causes superior survival in mice engrafted with systemic NPM-ALK^+^ ALCL

**Figure 5:**
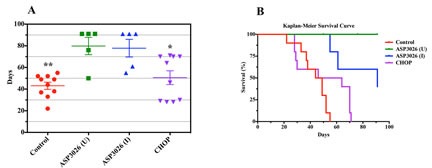
Treatment with ASP3026 is associated with superior overall survival in NPM-ALK^+^ ALCL (A) Scatterplot shows that the mean ± SE duration of survival in control mice was 43.1 ± 3.3 days. Mice treated with CHOP lived slightly longer (50.6 ± 6.4 days). In contrast, mice treated with uninterrupted ASP3026 (U) lived 79.8 ± 8.0 days and those treated with interrupted ASP3026 (I) lived 77.8 ± 8.1 days. Two mice treated with ASP3026 (U) died incidentally at 50 and 76 days because of infection, with no evidence of lymphoma tumors by imaging or by complete necropsy. In the ASP3026 (I) group, mice developed relapsed lymphoma after the interruption of ASP3026, and 2 mice were euthanized at 55 and 61 days because of extensive tumor burdens. The other 3 mice received ASP3026 after relapse and the lymphoma regressed. These mice survived until the termination of the study (*: *P* < 0.05; **: *P* < 0.01 vs. ASP3026 [U or I]). (B) Kaplan-Meier survival curves show that mice treated with ASP3026 (U or I) demonstrated statistically superior overall survival compared with control mice treated with vehicle or CHOP (*P* = 0.001 in ASP3026 [U] vs. control or CHOP; *P* = 0.001 in ASP3026 [I] vs. control; *P* = 0.04 in ASP3026 [I] vs. CHOP). Significance differences were not detected between control mice and mice treated with CHOP or between mice treated with ASP3026 (U) and mice treated with ASP3026 (I).

Whereas control mice survived 43.1 ± 3.3 (mean ± SE) days from study inception and mice treated with CHOP survived 50.6 ± 6.4 days, mice treated with ASP3026 (U) survived 79.8 ± 8.0 days, and those treated with ASP3026 (I) survived 77.8 ± 8.1 days (Fig. 5A). Kaplan-Meier survival curves (Figure 5B) show that mice treated with ASP3026 (U or I) had superior overall survival compared with mice treated with vehicle or CHOP.

## DISCUSSION

In the present study, we adopted comprehensive *in vitro* and *in vivo* experimental approaches that allowed in depth characterization of the effects of the recently developed ALK inhibitor ASP3026 in NPM-ALK^+^ ALCL. Our *in vitro* experiments demonstrated that ASP3026 causes a marked decrease in the viability of NPM-ALK^+^ ALCL cells, which could be at least partially explained by a significant increase in apoptotic cell death. Also, ASP3026 induces a significant decrease in cellular proliferation and anchorage-independent colony formation in NPM-ALK^+^ ALCL. Although all of the NPM-ALK^+^ ALCL cell lines used in this study demonstrated significant *in vitro* responses to ASP3026, some assays showed variable sensitivities of the different cell lines to the effects of ASP3026. Previous reports highlighted inherent biochemical differences between NPM-ALK^+^ ALCL cell lines that could explain their variable sensitivities to inhibitors of lymphoma cell survival [26].

When the direct effects of ASP3026 on NPM-ALK were examined, we found remarkable decreases in NPM-ALK tyrosine kinase activity and tyrosine phosphorylation levels at 2 residues, Y646 and Y664. It has been previously shown that Y664 is functionally relevant as it is the residue where NPM-ALK binds with survival-promoting proteins such as phospholipase C-γ and IGF-IR [27-29]. Downregulation of the phosphorylated form of NPM-ALK by ASP3026 was associated with a remarkable reduction in the phosphorylation levels of IGF-IR, STAT3, AKT, and JNK proteins, which are established targets of NPM-ALK signaling that contribute significantly to the survival of this lymphoma [28-33]. At the biochemical level, ASP3026 successfully induced cleavage of caspase 3 and PARP, further indicating that it induces apoptosis in NPM-ALK^+^ ALCL cells.

To analyze the effects of ASP3026 *in vivo*, we generated and utilized a systemic NPM-ALK^+^ ALCL model in SCID mice and monitored tumor development using a bioluminescence-based imaging system. Contrary to the widely utilized NPM-ALK^+^ ALCL xenograft models that exploit localized, subcutaneous tumor engrafts [34, 35], our approach is much more relevant to clinical settings where NPM-ALK^+^ ALCL patients typically present with widely disseminated disease. Uninterrupted oral administration of ASP3026 was associated with total eradication of established systemic lymphoma tumors with no evidence of relapse throughout the entire period of the study. In mice where ASP3026 was withheld after 2 weeks of daily administration, NPM-ALK^+^ ALCL tumors quickly relapsed, indicating that tumor suppression was secondary to the administration of ASP3026. Nonetheless, when treatment was resumed in some of these mice, lymphoma growth decreased significantly and the mice survived until study termination. In contrast to ASP3026-treated mice, mice treated with vehicle developed aggressive systemic lymphoma and had to be euthanized before study period ended. Importantly, we also evaluated the response of NPM-ALK^+^ ALCL to the commonly utilized CHOP regimen. Although the lymphoma initially regressed in response to CHOP, it eventually relapsed and mice had to be euthanized before study termination.

Histologic evaluation of NPM-ALK^+^ ALCL tumors in mice undergoing treatment with ASP3026 showed response to therapy in the form of much less proliferative lymphoma cells than those observed in control mice. Furthermore, immunohistochemical staining demonstrated that pSTAT3 and pIGF-IR expressions were significantly weakened in tumors collected after ASP3026 treatment, which supports the Western blotting data from the *in vitro* experiments.

NPM-ALK^+^ ALCL is an aggressive neoplasm. Although patients show a favorable response to CHOP, 30-40% of patients subsequently develop resistance, have multiple relapses, and frequently die. Factors that have been associated with poor outcome include stage III/IV disease at presentation, involvement of the mediastinum, viscera or skin, and elevated LDH levels [7, 12]. More recent studies have shown that the presence of specific molecular events at presentation or during therapy could distinguish subgroups of NPM-ALK^+^ ALCL patients with worse prognosis. For instance, detection of *NPM-ALK* transcripts in bone marrow or serum (i.e., minimal disseminated disease [MDD]) and levels of anti-NPM-ALK antibody in serum at diagnosis were used to stratify 128 patients into high-, intermediate- or low-risk groups. High- and intermediate-risk patients represented 20% and 31%, respectively, of this patient cohort. The progression-free survival rates were 28% and 68% in these 2 groups, respectively [36]. Furthermore, patients with MDD at diagnosis and minimal residual disease after their first course of CHOP-based chemotherapy showed an 81% cumulative incidence of relapse and only had a 65% 5-year overall survival rate [37]. These findings stress the urgent need to develop more specific and effective approaches to treat NPM-ALK^+^ ALCL.

Considering that the number of currently available ALK inhibitors is limited [38, 39], it is very important to study these inhibitors in different types of ALK^+^ tumors to determine their utility in clinical settings. Crizotinib is one of the most extensively studied ALK inhibitors; nonetheless, it has been more evaluated in ALK^+^ solid tumors [16-18]. Although crizotinib demonstrated excellent efficacy in eradicating EML4-ALK^+^ NSCLC tumors, recent reports have stressed the evolution of EML4-ALK mutations that are substantially resistant to crizotinib [21-23]. Indeed, several mechanisms, including somatic kinase domain mutations, *ALK* gene fusion copy number gain, and the emergence of separate oncogenic drivers, have been implicated in the initiation of crizotinib resistance [21-23, 40-43]. Resistance to crizotinib has also been reported in patients with inflammatory myofibroblastic tumor who presented with the BARBP2-ALK translocation and developed the ALK^F1174L^ activation mutation after treatment with crizotinib [44]. Notably, this mutation also resides in a subgroup of neuroblastoma patients, where it confers resistance to crizotinib [45]. More recently, resistance to crizotinib has also been described in NPM-ALK^+^ ALCL [46, 47]. Culturing NPM-ALK^+^ ALCL cell lines in increasing concentrations of crizotinib resulted in the evolution of 2 acquired mutants – NPM-ALK^I231N^ and NPM-ALK^L256Q^– that were remarkably resistant to crizotinib [46]. Furthermore, the NPM-ALK^I231N^ mutant was also identified in a lymphoma patient who was receiving crizotinib [47]. Our data show that ASP3026 effectively decreased the proliferation of 293T cells expressing these mutants. In addition, ASP3026 was capable of decreasing the phosphorylation of crizotinib-resistant NPM-ALK mutants at Y664. Considering that therapeutic resistance is a major hurdle for clinical utilization of crizotinib, these promising findings suggest that ASP3026 could be used to overcome crizotinib resistance in NPM-ALK^+^ ALCL.

In summary, we performed extensive *in vitro* and *in vivo* analyses of the effects of ASP3026 on the survival and growth of NPM-ALK^+^ ALCL. Our results provide strong evidence that ASP3026 could represent a novel and successful therapeutic strategy to eradicate this aggressive lymphoma and that patients with NPM-ALK^+^ ALCL, including those with crizotinib resistance, should be enrolled in the current clinical trials testing the effects of ASP3026 in ALK^+^ neoplasms.

## METHODS

### Cell lines

The NPM-ALK^+^ T-cell ALCL cell lines Karpas 299, SU-DHL-1, SUP-M2, SR-786, and DEL were purchased from DSMZ (Braunschweig, Germany). The 293T cells (ATCC, Manassas, VA) were used in transfection experiments. Human peripheral blood CD3^+^ pan-T lymphocytes were used in some experiments (catalogue number: PB009-1F) (StemCell Technologies, Vancouver, BC, Canada). The lymphoma cell lines and T lymphocytes were maintained in RPMI 1640 supplemented with 10% FBS, 2 mM glutamine, 100 U/mL penicillin, and 100 μg/mL streptomycin, and then incubated at 37°C in 5% CO_2_. 293T cells were maintained in DMEM under similar conditions.

### ALK inhibitor

ASP3026 (CT-ASP302; ChemieTek, Indianapolis, IN) was suspended in DMSO admixed with H_2_O and HCl (1:1) for *in vitro* experiments and in 0.5% methylcellulose for *in vivo* experiments.

### Antibodies

The following antibodies were purchased: pALK^Y1604^ (Y664 in NPM-ALK; 3341), pALK^Y1586^ (Y646 in NPM-ALK; 3343), pIGF-IR^Y1131^ (3021), pSTAT3^Y705^ (4113), AKT (9272), pAKT^S473^ (4051), JNK (3708S), pJNK^T183/Y185^ (4668S), PARP (9542P), cleaved PARP (5625S), and cleaved caspase-3 (9661S) (Cell Signaling Technology, Danvers, MA); pIGF-IR^Y1161^ (Ab39398) (Abcam, Cambridge, MA); IGF-IR (39-6700) (Invitrogen, Grand Island, NY); STAT3 (sc-8019) and caspase-3 (sc-7272) (Santa Cruz Biotechnology, Santa Cruz, CA); ALK (M719501-2) and Ki-67 (M7240) (Dako, Carpinteria, CA); and β-actin (A-2228) (Sigma-Aldrich, St. Louis, MO).

### MTS assay

Cell viability was measured using MTS reagent (3-(4,5-dimethylthiazol-2-yl)-5-(3-carboxymethoxyphenyl)-2-(4-sulfophenyl)-2H-tetrazolium; Promega, Madison, WI). Briefly, 1 × 10^5^ cells were seeded into 96-well plates, and 20 μL of MTS reagent was added to each well and incubated for approximately 2 h at 37°C in 5% CO_2_. Plates were read at 490 nm.

### *In vitro* apoptosis detection

Cytospin preparations were stained by Wright-Giemsa and analyzed by light microscopy for morphologic features consistent with apoptosis. Apoptosis was also detected using a commercially available kit (556547, BD Biosciences, San Jose, CA). Cells were stained with annexin V-FITC and propidium iodide (PI) and incubated at room temperature for 15 min in annexin binding buffer. Analysis was performed using flow cytometry (BD FACSCalibur) and CellQuest software (BD Biosciences).

### BrdU assay

Cell proliferation was measured using a standard kit (X1327K1, Exalpha Biologicals, Shirley, MD). Cells were plated at a concentration of 2 × 10^5^ cells/well after which 20 μL of BrdU (1:500 in cell culture medium) was added for 24 h. Plates were centrifuged for 5 min, fixed, and washed. Then, 100 μL of anti-BrdU antibody was added for 1 h, followed by 100 μL of peroxidase goat anti-mouse IgG conjugate (1:2000) for 30 min and then 100 μL of 3,3′,5,5′-tetramethylbenzidine (TMB) substrate for 30 min. Stop solution (50 μL) was added, and the plates were read at 450/595 nm using an ELISA plate reader.

### Anchorage-independent colony formation

Cells were treated with vehicle or ASP3026 for 48 h and then plated in methylcellulose-based medium (Methocult H4230; Stemcell Technologies) mixed in RPMI 1640 (1:5). Harvested cells were suspended in methylcellulose (v/v; 1:10) and poured into 24-well plates. The plates were incubated for 5 days at 37°C in 5% CO_2_. Colonies were stained using p-iodonitrotetrazolium violet and visualized using the FluorChem 8800 imaging system (Alpha Innotech, San Leandro, CA).

### Tyrosine kinase assay

NPM-ALK tyrosine kinase activity was measured using the Universal Tyrosine Kinase Assay Kit (Takara Bio, Pittsburg, PA). Briefly, 2 × 10^6^ cells were immunoprecipitated using standard protocol and ALK antibody (Dako) overnight at 4°C. Samples were spun and resuspended (including beads) in Kinase Reacting Solution and plated in triplicates in the protein tyrosine kinase substrate immobilized microplate (provided in the kit). ATP-2Na was added to each well in order to start tyrosine phosphorylation and incubated at 37°C for 30 min. The sample solution was then aspirated, and the wells were washed 4 times. Subsequently, samples were blocked with blocking solution for 30 min at 37°C, followed by addition of 50 μL of anti-phosphotyrosine (PY20)-horseradish peroxidase (HRP) solution for 30 min at 37°C. Finally, samples were washed and 100 μL of the HRP substrate TMB was added into each well for 15 min. The reaction was stopped by adding 100 μL of stop solution and absorbance was measured at 450 nm.

### Western blotting

The lysis buffer contained 25 mM HEPES (pH 7.7), 400 mM NaCl, 1.5 mM MgCl_2_, 2 mM EDTA, 0.5% Triton X-100, 0.1 mM phenylmethylsulfonyl fluoride, 3 mM dithiothreitol, phosphatase and protease inhibitor cocktails. Fifty to 80 μg total proteins were electrophoresed on 8% SDS-PAGE, transferred onto polyvinylidene fluoride membranes, and probed with primary antibodies and then matched secondary antibodies conjugated with HRP. Protein expression was detected using chemiluminescence and a commercially available kit (GE Healthcare, Piscataway, NJ).

### Site-directed mutagenesis

To generate the crizotinib-resistant NPM-ALK^I231N^ and NPM-ALK^L256Q^ mutants that are equivalent to ALK^I1171N^ and ALK^L1196Q^, a wild type NPM-ALK plasmid and the Quick Change II XL Site Directed Mutagenesis kit were used (Agilent Technologies, Santa Clara, CA) [28, 46, 47]. Primer sequences are depicted in Table 1. Five-μL of the PCR products were transformed using MaxEfficiency DH5α competent cells (Invitrogen), and the transformation reactions were plated in ampicillin resistant plates overnight at 37°C. Colonies containing the correct inserts, as determined by sequencing, were amplified overnight at 37°C in LB Broth containing ampicillin, and subjected to purification by using the Qiaprep Spin Miniprep Kit (Qiagen). Optical densities were acquired using spectrophotometry (NanoDrop 2000, Thermo Scientific, Waltham, MA).

**Table 1:** Primer sequences for crizotinib-resistant NPM-ALK mutants

NPM-ALK^I231N^: F, 5’-GGAAGCCCTGATCAATAGCAAATTCAACCACCAGAAC-3’;
R, 5’- GTTCTGGTGGTTGAATTTGCTATTGATCAGGGCTTCC-3’
NPM-ALK^L256Q^: F, 5’- CAATCCCTGCCCCGGTTCATCCTGCAGGAGCTCATGGCGGG-3’;
R, 5’- CCCGCCATGAGCTCCTGCAGGATGAACCGGGGCAGGGATTG-3’.

### Transfection

Briefly, 293T cells (1 × 10^6^) were plated in 6 well plates 24 h prior to transfection. Then, 2 μg of wild type NPM-ALK, NPM-ALK^I231N^ or NPM-ALK^L256Q^ were added to 100 μL OptiMEM medium. Simultaneously, 7 μL of Lipofectamine (Invitrogen) were added to 100 μL OptiMEM medium in a different tube. After incubation for 5 min at room temperature, the contents of the 2 tubes were admixed and incubated for 20 min at room temperature. The mixture was added to the corresponding well.

### Systemic xenograft NPM-ALK^+^ ALCL model

*In vivo* studies were preapproved by our Institutional Animal Care and Use Committee. Karpas 299 cells permanently expressing firefly luciferase and green fluorescence protein (GFP) were generated by using the F-Luc-GFP lentivirus (Capital Biosciences, Rockville, MD) in which humanized firefly luciferase (hLUC) was expressed under the CMV promoter. GFP and puromycin resistance marker were co-expressed bicistronically under the SV40 promoter [48]. Cells (1x10^6^/mouse) were injected into the tail veins of female C.B-17 SCID mice (6-8 weeks old; Taconic, Cambridge City, IN). Most of the mice developed NPM-ALK^+^ ALCL at approximately 3 weeks after injection. Lymphoma was monitored by luciferase signaling detection upon intraperitoneal injection of D-luciferin (Gold Biotechnology, St. Louis, MO) using bioluminescence imaging (IVIS Lumina XR imaging system; Caliper Life Sciences, Alameda, CA). ASP3026 (30 mg/kg daily) was administered using oral gavage at 3 weeks after injection of Karpas 299 cells*.* In one group (U), ASP3026 was administered uninterruptedly for 10 weeks, and in a second group (I), it was given for 2 weeks, interrupted for 4 weeks, and then continued for an additional 4 weeks. Control mice were given vehicle (0.5% methylcellulose). In a fourth group, CHOP [cyclophosphamide (40 mg/kg), doxorubicin (3.^3^ mg/kg), and vincristine (0.5 mg/kg) were given intravenously; and prednisone (0.2 mg/kg) was given by oral gavage] was administered for 5 consecutive days 3 weeks after injection of the lymphoma cell line [49, 50]. Tumor progression was monitored every week until death or until the study was concluded at week 13. The mice were euthanized and a total body necropsy was performed. Tumors were excised and fixed in 10% buffered formalin or snap-frozen in liquid nitrogen.

### *In vivo* apoptosis detection

Apoptosis detection in mice was performed by intraperitoneal injection of the firefly luciferase prosubstrate VivoGlo Caspase-3/7 (P1781; Z-DEVD-Aminoluciferin, Promega) and bioluminescence imaging (IVIS Lumina XR imaging system; Caliper Life Sciences).

### Immunohistochemical staining

Formalin-fixed and paraffin-embedded tissue sections from mouse lymphoma tumors were deparaffinized using an alcohol gradient. Sections were subsequently washed and subjected to antigen retrieval for 20 min in a steamer using 1× Target Retrieval Solution (Dako) and then allowed to cool down for 20 min to room temperature, washed, and incubated in 3% H_2_O_2_ for 15 min to block endogenous peroxidase activity. The sections were then blocked in serum-free blocking solution (Universal LSAB+ kit, Dako) for 30 min at room temperature. Primary antibody diluted in blocking buffer (1:50 for ALK and pSTAT3; 1:100 for pIGF-IR and Ki-67) was added for overnight incubation at 4°C. Next, the sections were washed 3 times and incubated with the secondary antibody LINK and then with secondary antibody streptavidin each for 30 min, and developed with 3,3′-diaminodbenzidine tetrahydrochloride substrate that includes HRP. Hematoxylin was used for counterstaining.

### Statistical analysis

Survival analysis was performed using PRISM software (GraphPad, La Jolla, CA) and the generation of Kaplan-Meier curves. The significance of differences between the groups was assessed using the Mantel-Cox log-rank test. For other data, statistical significance was detected by using one-way analysis of variance and the Bonferroni's post hoc multiple comparisons test. *P* < 0.05 was considered statistically significant.

### Authorship

S.K.G., D.V. participated in developing the conception of the study, designed experiments, performed research, analyzed data, and contributed to writing the paper; R.M. performed research; P.S. participated in developing the conception of the study, designed experiments, and analyzed data; H.M.A. developed the conception of and supervised the study, provided essential experimental tools, designed experiments, performed research, analyzed data, and wrote the paper. The authors declare no competing financial interests.
